# Characterization of Rotavirus Strains Responsible for Breakthrough Diarrheal Diseases among Zambian Children Using Whole Genome Sequencing

**DOI:** 10.3390/vaccines11121759

**Published:** 2023-11-26

**Authors:** Innocent Mwape, Natasha Makabilo Laban, Kennedy Chibesa, Andrew Moono, Suwilanji Silwamba, Moffat Mulemena Malisheni, Caroline Chisenga, Adriace Chauwa, Paul Simusika, Mabvuto Phiri, Michelo Simuyandi, Roma Chilengi, Corena De Beer, David Ojok

**Affiliations:** 1 Enteric Disease and Vaccine Research Unit, Centre for Infectious Disease Research in Zambia, Lusaka P.O. Box 34681, Zambia; natasha.laban@cidrz.org (N.M.L.); adriace.chauwa@cidrz.org (A.C.);; 2 Division of Medical Virology, Faculty of Medicine and Health Sciences, Stellenbosch University, P.O. Box 241, Cape Town 8000, South Africa; cdeb@sun.ac.za; 3 Department of Infection Biology, Faculty of Infectious and Tropical Diseases, London School of Hygiene and Tropical Medicine, London WC1E 7HT, UK; 4 Division of Medical Virology, School of Pathology, Faculty of Health Sciences, University of the Free State, Bloemfontein P.O. Box 339, South Africa; 5 Influenza Research Institute, University of Wisconsin-Madison, Madison, WI 53706-13380, USA; 6 University Teaching Hospitals, Lusaka 10101, Zambia; 7 Institute of Basic and Biomedical Sciences, Levy Mwanawasa Medical University, Lusaka 10101, Zambia

**Keywords:** rotavirus, vaccine, breakthrough, Zambia, children, reassortment

## Abstract

The occurrence of rotavirus (RV) infection among vaccinated children in high-burden settings poses a threat to further disease burden reduction. Genetically altered viruses have the potential to evade both natural infection and vaccine-induced immune responses, leading to diarrheal diseases among vaccinated children. Studies characterizing RV strains responsible for breakthrough infections in resource-limited countries where RV-associated diarrheal diseases are endemic are limited. We aimed to characterize RV strains detected in fully vaccinated children residing in Zambia using next-generation sequencing. We conducted whole genome sequencing on Illumina MiSeq. Whole genome assembly was performed using Geneious Prime 2023.1.2. A total of 76 diarrheal stool specimens were screened for RV, and 4/76 (5.2%) were RV-positive. Whole genome analysis revealed RVA/Human-wt/ZMB/CIDRZ-RV2088/2020/**G1**P[4]-I2-R2-C2-M2-A2-N2-T2-E2-H2 and RVA/Human-wt/ZMB/CIDRZ-RV2106/2020/**G12**P[4]-**I1**-R2-C2-M2-A2-**N1**-T2-**E1**-H2 strains were mono and multiple reassortant (exchanged genes in bold) respectively, whilst RVA/Human-wt/ZMB/CIDRZ-RV2150/2020/G12P[8]-I1-R1-C1-M1-A1-N1-T1-E1-H1 was a typical Wa-like strain. Comparison of VP7 and VP4 antigenic epitope of breakthrough strains and Rotarix strain revealed several amino acid differences. Variations in amino acids in antigenic epitope suggested they played a role in immune evasion of neutralizing antibodies elicited by vaccination. Findings from this study have the potential to inform national RV vaccination strategies and the design of highly efficacious universal RV vaccines.

## 1. Introduction

Group A rotaviruses (RVA) are responsible for 1.76 million hospitalizations among under five (U5) children worldwide, with a quarter of these cases occurring in Sub-Saharan Africa [1]. Rotavirus is a double-stranded RNA virus whose genome consists of 11 segments encoding six non-structural proteins (NSP1 to NSP6) and six structural viral proteins (VP1 to VP4, VP6 and VP7) [2,3]. A combination of VP4 and VP7 outer capsid proteins is used to classify RVA in G (VP7) and P (VP4) serotypes and/or genotypes [4]. G1P[8], G2P[4], G3P[8], G4P[8] and G9P[12] account for about 88% of RVA genotypes in humans globally [5]. Although the above binary system is traditionally used to classify RVA genotypes, the Rotavirus Classification Working Group (RCWG) has now recommended that the classification of RVA should be based on the whole genome constellation of all the 11 gene segments contained in the RVA genome. For example, Gx-P[x]-Ix-Rx-Cx-Mx-Ax-Nx-Tx-Ex-Hx, where x denotes the genotype number and the letter represents the name of the gene [6]. Whole genome sequencing (WGS) studies are important for monitoring RVA genetic diversity, which is mainly driven by the error-prone viral RNA-dependent RNA (RdRNA) polymerase [7], with most human RVA strains showing high similarity to the Wa-like genogroup 1 constellation (Gx-P[x]-I1-R1-C1-M1-A1-N1-T1-E1-H1) or the DS-1-like genogroup 2 constellation (Gx-P[x]-I2-R2-C2-M2-A2-N2-T2-E2-H2). A few human RVA strains have been categorized in the Au-like genogroup 3 (Gx-P[x]-I3-R3-C3-M3-A3-N3-T3-E3-H3) [8]. Furthermore, WGS has provided evidence supporting genetic reassortment between different RVA genogroups and interspecies transmission of the segments of the viral genome [6,9]. Since neutralizing antibodies are mainly elicited against the outer viral proteins, genetic alterations in the RVA genome spearheaded by either the error-prone RdRNA polymerase or reassortment of the VP7 and VP4 gene-carrying segments might modify antibody protection against RVA gastroenteritis [4]. Therefore, monitoring the evolution of RVA using WGS represents a useful tool for evaluating vaccine effectiveness [6,9].

Vaccination is the most cost-effective way to control and prevent infectious diseases. The World Health Organization (WHO) approved RVA vaccines including Rotarix (GlaxoSmithKline, Brentford, UK), Rotateq (Merck, Rahway, NJ, USA), Rotavac (Bharat Biotech, Turakapally, India) and Rotasiil (Serum Institute of India, Pune, India). Rotarix and Rotavac are monovalent vaccines comprising G1P[8] and G9P[11] human RVA strains, respectively, while Rotateq and Rotasiil contain five pentavalent RVA strains, G1, G2, G3, G4 and P[8] and G1, G2, G3, G4 and G9, respectively [10]. The RVA vaccine employed in Zambia since 2012 is Rotarix, with modest seroconversion rates of approximately 60% [11]. A small percentage of children who have received all recommended doses (fully vaccinated) of RVA vaccines can sometimes be reinfected [12,13,14] with vaccine and non-vaccine RVA strains [15,16,17]. We defined all RVA infections occurring in children after full vaccination as breakthrough infections. Breakthrough infections are mainly caused by genetically altered (variant) strains [18]. Variant strains may exhibit increased transmission capabilities, increased virulence, and/or pathogenicity, leading to higher hospitalization, morbidity, and mortality rates, and may acquire the ability to escape both natural and vaccine-induced immunity [18]. Although the occurrence rate of RVA breakthrough infections might seem low, the threat to public health and the economy, especially in endemic regions, cannot be emphasized enough. Characterizing RVA variants causing breakthrough infections using WGS has significant implications for vaccination strategies and the design of highly effective next-generation RVA universal vaccines. Additionally, the high diarrheal burden in LMIC justifies conducting this kind of study in these settings.

## 2. Materials and Methods

### 2.1. Study Participants

**Figure 1:**
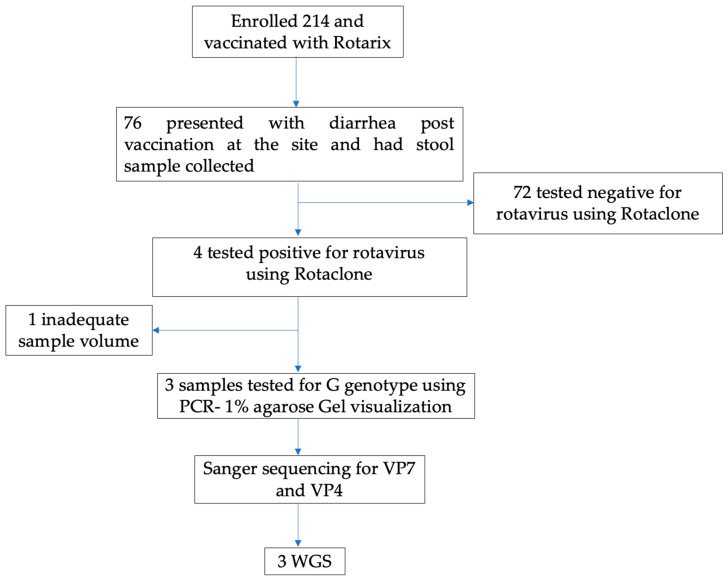
Study flow Chart.

We used stool samples collected and tested for rotavirus under a rotavirus vaccine clinical trial conducted in Lusaka, Zambia. The trial was registered under the Pan African Clinical Trials Registry number PACTR201804003096919. This trial evaluated the safety and immunogenicity benefits of an additional third dose of Rotarix vaccine administered at 9 months of age [19]. Briefly, 214 infants aged 6 to 12 weeks were enrolled from 13 September 2018 to 15 November 2018 and followed up to 3 years of age. All infants received the routine two doses of the Rotarix vaccine and, at 9 months old, were randomized to receive a third dose of the Rotarix vaccine concomitantly with measles-rubella vaccination (intervention arm) or receive only measles-rubella vaccine (control arm). In the first year of follow-up, 76 infants presented to the study clinic with acute diarrhea and had a stool sample collected and stored at −20 °C prior to testing. A total of 4/76 (5.2%) stool specimens were rotavirus-positive by an enzyme immunoassay, Premier™ Rotaclone^®^ (Meridian Bioscience, Inc., Cincinnati, OH, USA). Of these, three with sufficient stool specimens available for molecular analysis were identified as G genotypes, two G3, and one G4 genotype, using VP7-based polymerase chain reaction (PCR) assay on 1% agarose gel visualization stained with gel red (Biotium, CA, USA) [19]. In this study, we performed Sanger Sequencing of the VP7 and VP4 of these three RV infections to obtain G and P genotypes, respectively, followed by WGS using the Illumina Miseq platform, as shown in Figure 1.

### 2.2. Laboratory Procedures

#### 2.2.1. Rotavirus Double-Stranded RNA Extraction

The rotavirus RNA was extracted from 140 μL of the 10% stool suspension using the Qiagen viral RNA mini kit as per the manufacturer protocol (Qiagen GmbH, Hilden, Germany) [20].

#### 2.2.2. Sanger Sequencing of VP7 and VP4

The reverse transcription PCR (RT-PCR) was performed with primers adapted from the previously published protocol [21] (Supplementary Data). Pipetted 5 μL of extracted RNA were denatured at 95 °C for 5 min. Thereafter, the RT-PCR was performed using One step SuperScript III with Platinum Taq High fidelity DNA polymerase kit (Invitrogen, Waltham, MA, USA) as follows: (5 μL denatured RNA, 1 μL reverse primer (10 μM), 1 μL forward primer (10 μM), 25 μL 2X reaction mix, 1 μL enzyme mix and 17 μL of H_2_O). The reverse transcription reaction was carried out at 50 °C for 30 min. The denaturation of reverse transcriptase and activation of the platinum Taq occurred at 94 °C for 2 min with subsequent 40 cycles amplification with the following thermocycling conditions: (15 s at 94 °C, 30 s at 45 °C, 3.5 min at 68 °C for VP4 and 1.5 min at 68 °C for the VP7). The PCR products were visualized at 1% agarose gel stained with gelred (Biotium, Fremont, CA, USA). The amplicons were purified using the GeneJet purification kit according to the manufacturer protocol (ThermoFisher Scientific, Waltham, MA, USA). The termination cycle sequencing reaction was carried out using the BigDye terminator V3.1 cycle sequencing kit and amplification primers were used in sequencing. Post-termination reaction, the products were purified, then dissolved in HI-DI™ Formamide (Thermo Fisher Scientific, Waltham, MA, USA), and loaded on an ABI 3130xl genetic analyzer. The sequence chromatograms were analyzed using Sequencher v5.0 (Gene Codes Corporation, Ann Arbor, MI, USA). The genotypes were determined using both BLASTn on NCBI and the BVR-BC database.

#### 2.2.3. Whole Genome Amplification

The Qiagen one-step RT-PCR kit was used to synthesize the cDNA with further amplification according to the previous protocol [22] (Supplementary Data). A 5 μL volume of extracted RNA, as well as the RV gene-specific primers from previously published methods [22,23], were incubated at 95 °C for five minutes. Thereafter, reverse transcription reaction occurred at 45 °C for 30 min, with subsequent 40 cycles with the following thermocycling conditions at 94 °C for 10 s, annealing at 55 °C for 1 min and extension at 68 °C for 3 min with final extension at 68 °C for 10 min. The PCR products were visualized on the 1% agarose gel stained with gel red (Biotium, CA, USA). Post-amplification products were purified using the GeneJet PCR purification kit as per the manufacturer’s protocol (ThermoFisher Scientific). The purified amplicons were quantified on the qubit version 3 using the high-sensitivity DNA reagents (Invitrogen).

#### 2.2.4. Library Preparation and Sequencing

The library preparation was performed according to the Illumina DNA Prep protocol (Illumina, San Diego, CA, USA) [24]. The quantified DNA was first normalized and then added to the mixture of tagmentation reagents. The mixture was incubated on a thermocycler for 15 min at 55 °C. Furthermore, the post-tagmentation cleanup was performed by stopping the tagmentation reaction by adding the stop tagmentation buffer with further incubation at 37 °C for 15 min. The tagmented DNA was cleaned up by adding the tagmentation wash buffer to the mixture. Further, the amplification of the tagmented DNA was performed by the addition of the index (i7), index (i5), and oligos required for the cluster generation, as well as the addition of the enhanced PCR mix reagent. The amplified tagmented fragments of the library were purified on the magnetic stand using ethanol and Illumina purification beads (IPB). The purified library was further quantified on the Qubit 3.0 fluorometer (ThermoFisher Scientific, Waltham, MA, USA) and then normalized. The normalized library was then diluted and denatured to the final loading concentration to the v2 500 cycle Miseq reagent kit.

#### 2.2.5. Genome Assembly

Post-sequencing from the Illumina MiSeq, paired-end reads were imported into Geneious Prime version 2023.1.2 The FASTQ files were first trimmed using the BBDuk (Bestus Bioinformaticus Decontamination using Kmers), a plugin in the Geneious Prime software [25]. The trimmed sequence reads were assembled using both the de novo and mapped to reference sequences [21]. Lastly, the consensus sequences were extracted and used for the downstream analysis.

#### 2.2.6. Determination of Genotype

The genotype for each of the 11 segments of the genome was determined by utilizing the Bacterial and Viral Bioinformatics Resource Center (BV-BVR), an online database that houses ViPR (Viral Pathogen Resources) [26]. Additionally, two online databases were used as compensatory methods in the genotype identification for each segment of the genome: (i) the Basic Local Alignment Search Tool (BLAST) on NCBI as well as an online (ii) rotavirus genotyping tool version 0.1 [27,28].

#### 2.2.7. Phylogenetic Analysis

For each of the 11 segments of the rotavirus genome, multiple reference sequences were obtained from both NCBI using the BLAST tool as well as the BV-BVR database. The multiple sequence alignment was performed using MAFFT, a plugin in Geneious Prime 2023.1.2, as well as MUSCLE in MEGA 6 [25,29]. The maximum likelihood trees for each of the 11 segments of the genome were constructed based on the generated substitution models in MEGA 6 using 1000 bootstraps. The model with the lowest BIC (Bayesian Information Criterion) value was selected for the phylogenetic tree construction [30,31]. The models used to build the phylogenetic trees in this study were: T92+G (VP1, VP2, VP3, VP4, VP7, NSP4, and NSP5), and T92+G+I (NSP1, NSP2, NSP3, and VP6).

## 3. Results

### 3.1. Baseline Characteristics as Well as Genotyping of VP4 and VP7

As shown in Table 1, the three rotavirus infections from infants RV2088, RV2106, and RV2150 were identified as G1P[4], G12P[4], and G12P[8] genotypes, respectively, using the VP7 and VP4 sequences obtained from sanger sequencing. Children among whom these infections occurred had a mean age of 22.3 months; two out of three were male, and only one was HIV exposed. Two infants had received three doses of the Rotarix vaccine and one had received two doses. Based on a fold increase in RV-specific immunoglobulin A (RV-IgA) titer between baseline and one month after the second dose of Rotarix, two infants (RV2088 and RV2150) had seroconverted, and one (RV2106) had not seroconverted.

### 3.2. Whole Genome Genotype Constellation

Using the RCWG classification, we detected a mono reassortant strain for RV2088 denoted as RVA/Human-wt/ZMB/CIDRZ-RV2088/2020/**G1**P[4]-I2-R2-C2-M2-A2-N2-T2-E2-H2 and a multiple reassortant strain for RV2106 denoted as RVA/Human-wt/ZMB/CIDRZ-RV2106/2020/**G12**P[4]-**I1**-R2-C2-M2-A2-**N1**-T2-**E1**-H2. The two reassortants had mostly the DS-1-like genetic backbone with Wa-like exchanged genes bolded. A typical Wa-like strain was detected for RV2150 denoted as RVA/Human-wt/ZMB/CIDRZ-RV2150/2020/G12P[8]-I1-R1-C1-M1-A1-N1-T1-E1-H1. For each of the 11 segments of the genome of the study strains complete or near complete lengths of the open reading frame (ORFs) were obtained (Table 2). The ORFs of sequences for all 11 genes of the breakthrough strains were deposited in the GenBank under the accession numbers (OR338245-OR338277).

### 3.3. VP7 Phylogenetic Analysis

**Figure 2:**
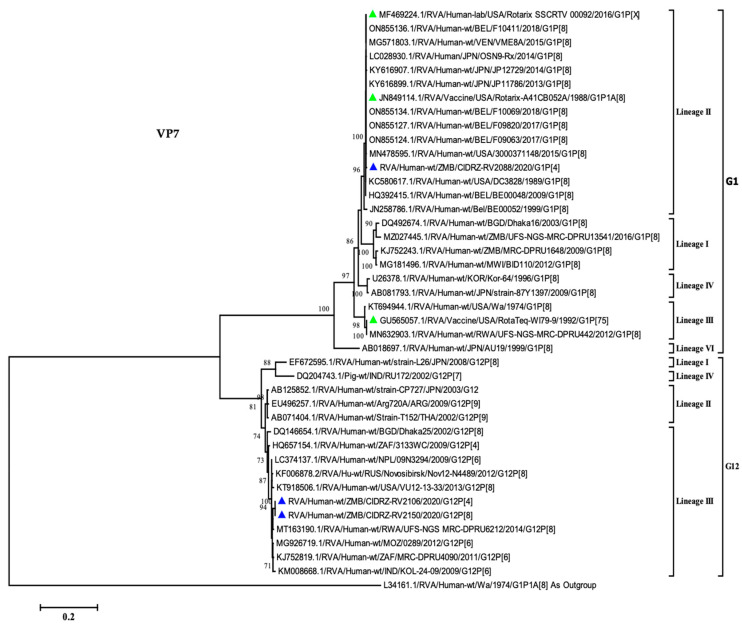
Maximum likelihood phylogenetic tree between the VP7 gene of the breakthrough G1 and G12 strains as well as global strains. Green-filled triangles represented vaccine sequences, whilst breakthrough strains were represented by blue-filled triangles. The scale at the bottom indicates nucleotide substitutions per site, whereas bootstrap values ≥ 70 were shown on the branch nodes.

In order to comprehend the relationship between the breakthrough strains and global strains, the phylogenetic tree was constructed for the VP7 gene of rotavirus. As shown in Figure 2, the G1 breakthrough strain clustered in lineage II along with the Rotarix vaccine as well as the designated lineage II reference strains [16,21,32]. The breakthrough strain CIDRZ-RV2088/2020/G1P[4] shared 99.7% nucleotide identity with the Rotarix vaccine, as well as vaccine-derived strains from Japan (KY616899.1), Belgium (ON855136.1) and USA (MF469224.1). Furthermore, the phylogenetic tree analysis shows that the two G12 strains clustered in lineage III, which comprised strains from Africa, the USA and Asia [32,33]. The two G12 strains exhibited 98% to 99.08% nucleotide identity with strains from Mozambique (MG926719.1), Rwanda (MT163190.1), South Africa (KJ752819.1) and Nepal (LC374137.1). The G12 strains (CIDRZ-RV2150/2020/G12P[4] and CIDRZ-RV2106/2020/G12P[8]) shared the nucleotide identity of 100% (Supplementary Data, Data S1).

### 3.4. Comparative Analysis of VP7 Antigenic Epitope between the Vaccines and Breakthrough Strains

**Figure 3:**
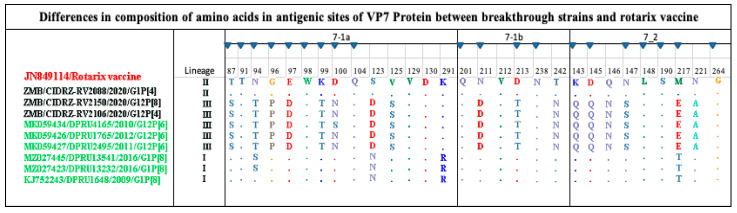
Amino acid alignment of the antigenic sites of the G1 and G12 strains are highlighted in black, reference strains green and red for the Rotarix vaccine. Blue-filled triangles represented amino residue positions that have been shown to escape neutralization with monoclonal antibodies. Dots represented conserved amino acids.

The VP7 forms part of the outer RV protein capsid, which is involved in eliciting the neutralizing antibodies [34]. The VP7 antigenic epitope comprises two domains, the 7-1 and 7-2. The 7-1 is further subdivided into 7-1a and 7-1b. The VP7 antigenic epitope is composed of 29 amino acids [35]. As shown in Figure 3, the CIDRZ-RV2088/2020/G1P[4] strain shared all the 29 amino acid residues of the Rotarix vaccine. Analysis of the antigenic epitope of the CIDRZ-RV2150/2020/G12P[4] and CIDRZ-RV2106/2020/G12P[8] strains revealed 12 conserved amino acids out of 29 in comparison to the Rotarix vaccine. The G12 strains exhibited eight amino acid differences in the 7-1a (T87S; N94T; G96P; E97D; K99T; D100N; S123D and V125S), whereas in 7-1b, three amino acid differences were observed (N211D; D213T and T242N). Furthermore, six amino acid differences were observed in the 7-2 (K143Q; D145Q; Q146N; N147S; M217E and N221A).

### 3.5. Phylogenetic Analysis of VP4 Gene

**Figure 4:**
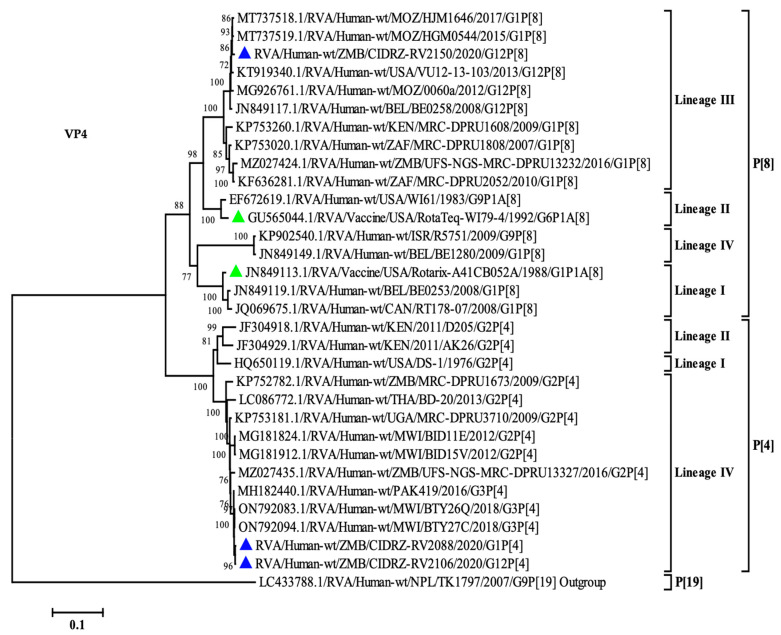
Maximum likelihood phylogenetic tree between the VP4 gene of the breakthrough P[8] and P[4] strains as well as global strains. Green-filled triangles represented vaccine sequences, whereas blue-filled triangles represented breakthrough strains. The scale at the bottom indicates nucleotide substitutions per site, whilst bootstrap values ≥ 70 were shown on the branch nodes.

To determine the relationship of the VP4 gene study strains, a phylogenetic tree was built, which contained global strains from previously published studies [21,36,37,38]. The P[4] strains (CIDRZ-RV2088/2020/G1P[4] and CIDRZ-RV2106/2020/G12P[4]) belonged to lineage IV, along with strains from Malawi, Uganda, Pakistan, and Thailand as shown in Figure 4. The P[4] breakthrough strains possessed nucleotide identities of 99.6% with strains from Malawi (ON792094.1, ON792083.1) and Pakistan (MH182440.1). See Supplementary Data, Data S2. Furthermore, the strains shared nucleotide identities of 99.8%. On the other hand, strain CIDRZ-RV2106/2020/G12P[8] belonged to lineage III together with strains from Africa, the USA, and Europe. The P[8] strain shared 99% nucleotide identities with human rotavirus strains from Mozambique (MT737518.1, MT737519.1) and USA (KT919340.1) (Supplementary Data, Data S2). Furthermore, the P[8] strain shared a nucleotide identity of 90% with the P[8] Rotarix vaccine (Supplementary Data, Data S2).

### 3.6. Comparative Analysis of VP4 Neutralizing Antigenic Epitope between Rotarix Vaccine and Breakthrough Strains

**Figure 5:**
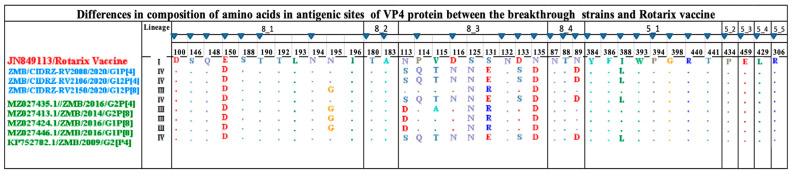
Amino acid alignment of the antigenic sites of the Rotarix vaccine highlighted in red, P[4] and P[8] breakthrough strains highlighted in blue and reference strains. Blue-filled triangles represented amino residues that have been shown to escape neutralization with monoclonal antibodies. Dots represented conserved amino acids.

The VP4 spike protein is proteolytically cleaved into the two distinctly functional proteins of the VP5* and VP8* [21]. The VP8 epitope is composed of four subdomains; 8-1, 8-2, 8-3, and 8-4. On the other hand, the VP5 is composed of five subdomains: 5-1, 5-2, 5-3, 5-4 and 5-5 [21]. The combination of antigenic epitopes of VP8 and VP5 is composed of 37 amino acids. In the present study, we report 25 conserved amino acids out of 37 in all breakthrough strains in comparison to the Rotarix see Figure 5. The breakthrough strains with the P[4] genotype had one amino acid difference in the 8-1 (E150D); further, in subdomain 8-2, all the amino acids were conserved, in subdomain 8-3 there were eight observed amino acid differences (N113S, P114Q, V115T, D116N, S125N, S131E, D133S and N135D). Lastly, in subdomain 8-4, only one amino acid difference was observed, N89D. The VP5 subunit of spike protein comprised five subdomains with the composition of 12 amino acids, of which only one amino acid difference, I388L, was observed. The comparison of the P[8] breakthrough strain with the Rotarix vaccine revealed two amino acid substitutions in the 8-1 subdomain (E150D and N195G). In the 8-3 subdomain, three amino acid substitutions were observed (S125N, S131R, and N135D). Finally, in the VP8* subdomain 8-4, all the amino acids were conserved. The VP5* subunit of the spike protein of the breakthrough strain P[8] revealed that all amino acids were conserved.

### 3.7. Phylogenetic Analysis of VP1, VP2, VP3 and VP6

The VP1 gene revealed the Wa-like study strain CIDRZ-RV2150/2020/G12P[8] belonged to lineage R1 along with strains from South Africa, Mozambique, Kenya, Belgium and Asia (Supplementary Figures, Figure S1). The Wa-like breakthrough strain displayed 98.7% homology with strains from South Africa (KJ752339.1), Malawi (MG181480.1), Bangladesh (DQ146649.1), (Supplementary Data, Data S3). Furthermore, the two DS-1-like strains clustered in lineage R2 together with the Zambian strain from 2015. The two DS-1-like breakthrough strains shared 98–99% nucleotide identity along with 2015 Zambian strains (OQ133150.1) as well as strains from Malawi (ON792025.1) and Pakistan (MH166402.1) (Supplementary Data, Data S3).

Phylogenetically, the VP2 gene revealed that the Wa-like strain CIDRZ-RV2150/2020/G12P[8] belonged to lineage C1 along with strains from South Africa, Mozambique, Kenya, USA and Asia (Supplementary Figures, Figure S2). The Wa-like breakthrough strain shared a nucleotide identity of 98-99% with strains from Mozambique (MT737433.1), Italy (KU048553.1), Rwanda (MN633028.1), and South Africa (MT855252.1) (Supplementary Data, Data S4). The DS-1-like strains belonged to lineage C2 and shared 98–99% nucleotide homology with strains from Malawi (ON792114.1) and Pakistan (MH166409.1) (Supplementary Data, Data S4).

The VP3 segment of the Wa-like breakthrough strain phylogenetically belonged to lineage M1 together with strains from India, Europe, and the USA (Supplementary Figures, Figure S3). The Wa-like study strain shared the 99% nucleotide identity with strains from Mozambique (MT737465.1), India (MN066756.1), and Italy (KU048583.1) (Supplementary Data, Data S5). Whereas the DS-1-like strains clustered in lineage M2 along with strains from Malawi, Pakistan, India, and the USA with shared nucleotide identities ranged from 98–99% (Supplementary Data, Data S5).

Phylogenetic analysis of VP6 gene has shown breakthrough strains (CIDRZ-RV2106/2020/G12P[4] and CIDRZ-RV2088/2020/G1P[4]) clustered in lineage I2 along with strains from Asia and Africa although strain CIDRZ-RV2106/2020/G12P[4] clustered separately from the other strains (Supplementary Figures, Figure S4). This strain shared the highest nucleotide identity of 91.8%, with a strain from Rwanda (MT163203). Whereas strain (CIDRZ-RV2088/2020/G1P[4]) displayed the highest nucleotide identity of 97.9% with a strain from Malawi (ON791997.1), Pakistan (MH170023.1), and Russia (KC713896.1). Furthermore, the Wa-like strain belonged to the I1 lineage and shared 99% nucleotide similarities with strain from Mozambique (MT737536.1) and India (MN067062.1) (Supplementary Data, Data S6).

### 3.8. Phylogenetic Analysis of NSP1, NSP2, NSP3, NSP4 and NSP5

Phylogenetically, the Wa-like strain belonged to lineage A1 along with strains from East and Southern Africa, as well as strains from Nepal and the USA see Supplementary Figures, Figure S5. The Wa-like breakthrough strain shared the nucleotide identity of 99% with strains from Mozambique (MT737605.1), Kenya (MZ097119.1), South Africa (MT854759.1) and Nepal (LC374132.1). On the other hand, the DS-1-like strains belonged to lineage A2 and exhibited the highest nucleotide identity of 97% with human rotavirus strains from Malawi (ON792097.1, ON792020.1, and ON791988.1). See Supplementary Data, Data S7.

Phylogenetic analysis of the NSP2 gene of CIDRZ-RV2150/2020/G12P[8] strain revealed the Wa-like study strain belonged to lineage N1 along with strains from Mozambique, Malawi, Kenya, Japan, and India, with highest shared nucleotide identity of 99.3% with a strain from Mozambique (MT737644.1) (Supplementary Data, Data S8 and Supplementary Figures, Figure S6). On the other hand, the DS-1-like breakthrough strains clustered in lineage N2. Furthermore, strain CIDRZ-RV2106/2020/G12P[4] clustered separately in lineage N2 and showed the highest nucleotide identity of 94% with strains from Malawi (ON792109.1 and ON792087.1) and Pakistan (MH182456.1) (Supplementary Data, Data S8).

Phylogenetically, the Wa-like strain belonged to lineage T1 and shared the highest nucleotide homology of 99% with strains from Mozambique (MT737680.1 and MT737699.1) (Supplementary Data, Data S9 and Supplementary Figures, Figure S7). Furthermore, the DS-1-like strains clustered in lineage T2 along with strains from Malawi, Pakistan and the USA. Additionally, strain CIDRZ-RV2106/2020/G12P[4] was separately grouped in lineage T2 and shared the maximum nucleotide identity of 91% with strains from Malawi (ON792110.1, ON792099.1) and Pakistan (MH182461.1). See Supplementary Data, Data S9, and Supplementary Figures, Figure S7.

The Phylogenetic analysis of the NSP4 gene has shown the breakthrough strains (CIDRZ-RV2106/2020/G12P[4] and CIDRZ-RV2150/2020/G12P[8]) clustered in lineage E1, along with strains from Africa, Asia and Brazil. Furthermore, strain CIDRZ-RV2106/2020/G12P[4] clustered separately in the lineage E1 from other strains. It shared the highest nucleotide identity of 96% with strains from Mozambique (MT737707.1), South Africa (MT854762.1), and Brazil (MF161827.1) (Supplementary Data, Data S10 and Supplementary Figures, Figure S8).

Phylogenetically, the NSP5 gene of Wa-like strain clustered in the lineage H1 along with strains from Europe, Asia, USA, Brazil, and Africa with the highest shared nucleotide identity of 99% with strains from South Africa (KP752581.1), Brazil (KU361041.1) and Spain (MH171474.1) (Supplementary Data, Data S11, and Supplementary Figures, Figure S9). On the other hand, the DS-1-like breakthrough strains belonged to lineage H2 with the highest nucleotide similarity of 99.7% with strains from Malawi (ON792112.1), South Africa (KJ753786.1), Ghana (LC105587.1) Pakistan (MH182471.1).

## 4. Discussion

We aimed to characterize the rotavirus strains responsible for breakthrough infections using samples from a cohort of children under five years old in Zambia. Rotavirus strains causing breakthrough diarrheal diseases continue to pose an obstacle to further the reduction of the disease burden [14].

The rotavirus strains detected in breakthrough infection were mono, multiple reassortants, and the typical Wa-like strain. The mono reassortant rotavirus infection suggests the genetic exchange occurred between the Rotarix vaccine VP7 gene and the wild-type DS-1-like strain. The VP7 gene of the mono reassortant G1P[4] was absolutely identical to the Rotarix vaccine, as well as the Rotarix vaccine-derived strains from Japan (KY616899.1), Belgium (ON855136.1) and the USA (MF469224.1) with shared nucleotide identity of 99.7%. A duration of 323 days had elapsed from the last receipt of the Rotarix vaccine to the detection of rotavirus. Therefore, the time elapsed suggested the infant was not shedding the Rotarix vaccine, as the reported duration of shedding the vaccine was mainly 6–20 days following the last vaccination, with a peak at the seventh day [39]. Therefore, the observed vaccine reassortant suggested to have been acquired from community circulation and not the result of vaccine shedding. This finding is consistent with studies from the USA and Belgium, where vaccine-reassortants were detected in infants with acute gastroenteritis [15,17].

Additionally, other studies have demonstrated that both vaccine-derived and vaccine-reassortant strains are generated due to genetic exchange between the vaccine and wild-type strain [16,40]. Clinical trial studies conducted in Malawi and South Africa on the Rotarix vaccine demonstrated that the vaccine induced both the homotypic and heterotypic neutralizing antibodies response, which protected children against the development of severe acute gastroenteritis; however, it did not protect against reinfection [41]. On the other hand, the analysis of the multiple reassortant strain revealed the exchange of four genes between the Wa-like and DS-1-like strains, indicating an intergenogroup exchange of genetic material. The multiple reassortments of genes between the Wa-like and the DS-1-like have previously been reported from a study in Rwanda and Vietnam in the post-vaccination era [42,43].

Phylogenetic analysis of the VP7 gene demonstrated the G12 strains clustered in lineage III along with other global strains. This is consistent with the finding by Rakau and colleagues, which demonstrated that most African G12 genotypes clustered in lineage III [34]. Furthermore, the G12 strains displayed high sequence homology with strains from Mozambique (RVA/Human-wt/MOZ/0289/2012/G12P[6], Rwanda (RVA/Human-wt/RWA/MRC-DPRU8020/2014/G12P[8] and South Africa (RVA/Human-wt/ZAF/MRC-DPRU4090/2011/G12P[6] which indicated local and regional transmission [42,44]. Phylogenetic analysis of the VP4 gene revealed the P[4] strains belonged to lineage IV along with strains from Malawi, Uganda and Pakistan [38,45]. The P[4] breakthrough strains shared a high homology of 99% with human rotavirus strains from Malawi, further indicating local and regional circulation. The P[8] breakthrough strain clustered in lineage III distantly from the P[8] Rotarix vaccine clustered in lineage I. The P[8] strain was closely related to strains from Mozambique with a nucleotide identity of 99%, furthermore indicating the transmission between two neighboring countries. Phylogenetically, four segments (VP6, NSP2, NSP3 and NSP4) of strain (CIDRZ-RV2106/2020/G12P[4]) clustered separately from the global strains. In each of the phylogenetic trees, the branching node was supported by strong bootstraps of 73%, 99%, 99%, and 77%, respectively. This phenomenon of certain genes clustering independently from other strains in the lineage was recently reported in the study conducted in Zambia by Nyaga and colleagues assessing a strain that had two segments clustering separately from the other strains in the lineages [37]. It remained unclear to what extent the four distinct clustered genes influenced rotavirus infectivity as this child did not show any seroconversion to the G1P[8] genotype of the Rotarix vaccine.

The outer capsid proteins of rotavirus, the VP4 and VP7, elicited neutralizing antibodies that targeted the antigenic epitope. Therefore, differences in the amino acids between the circulating strains and the vaccine strain in the antigenic epitope might affect the vaccine-mediated immune protection [21]. The VP7 antigenic epitope of the breakthrough strains was compared with the cognate epitope of the Rotarix vaccine. All the VP7 antigenic epitopes of G1 breakthrough strain amino acids were conserved in comparison to the Rotarix vaccine. However, the G12 strains had several amino acid differences in comparison to the Rotarix vaccine, which included radical differences such as G96P and M217E. Furthermore, in the observed radical variation M217E, methionine was hydrophobic, whereas the G12 strains contained glutamate, which is negatively charged [46]. The increased number of amino acid differences observed might affect the binding of the vaccine-elicited neutralizing antibodies against the G12 breakthrough strains. Our finding was consistent with a previous study from the USA, where the G12 genotype was predominant among the vaccinated children with acute gastroenteritis, and their comparison between the VP7 component of the vaccine and USA G12 strains revealed several amino acid differences in the antigenic epitope [47].

Rotavirus-infected mature enterocytes via the proteolytically cleaved VP4 by trypsin into functional subunits the VP5* and VP8* [21]. Due to the lower variability in the VP4, it has been shown to be the component of the vaccine involved in the heterotypic immune response [48]. The comparison of the P[4] breakthrough strains and VP4 of vaccine components revealed two amino acid differences that were radical in nature (P114Q and V115T). The P114Q was an immune escape mutation where radical variation was observed, in that the Proline contained in the vaccine was nonpolar compared to the polar glutamate contained in the breakthrough strains. Furthermore, the hydrophobic valine contained in the vaccine was different from the breakthrough strains, which had small polar threonine [46]. The non-conservative variations observed might affect the binding of the vaccine-elicited antibodies on the antigenic epitopes of the study strains. It has been shown from a previous study that the neutralizing antibodies generated by the VP4 vaccine with the presence of a single VP4-specific antibody to a specific epitope were sufficient to achieve neutralization in vitro [48]. Therefore, the conserved amino acids observed in immune escape positions in this study might play a role in the protection against gastroenteritis. A notable strength of our study was that the study was conducted in children with known vaccination history, seroconversion and time to diagnosis of infection post-vaccination. Our study had limitations, such as the inability to perform neutralization assays on the rotavirus strains causing the breakthrough infections and detect the role of other enteropathogens in the cause of diarrhea in the children. Furthermore, the four cases of rotavirus were detected only from the children who presented with diarrhea at the health facility based on the passive surveillance; therefore, it is possible that other cases could have occurred but were missed by this kind of surveillance. Additionally, during screening, some of the cases might have been missed due to the lower sensitivity of EIA as compared to the RT-PCR assay.

## 5. Conclusions

Our data shows the importance of post-market surveillance of vaccines in identifying rotavirus strains responsible for breakthrough infections and monitoring viral evolution in order to inform the next generation of vaccines. We report mono and multiple reassortant strains implicated in breakthrough infections. Furthermore, differences observed in amino acids in the VP7 and VP4 antigenic epitope at immune escape positions might have played a role in the evasion of antibodies elicited by the vaccine.

## Figures and Tables

**Table 1:** Baseline factors and immune characteristics of infants with breakthrough rotavirus infection.

	RV2088	RV2106	RV2150
Genotype	G1P[4]	G12P[4]	G12P[8]
Age (Months)	22	22	23
Sex	male	female	male
HIV exposure	unexposed	exposed	unexposed
Seroconversion	yes	no	yes
Number of doses received	three	three	two

**Table 2:** Whole genome constellation of breakthrough strains.

ID		VP7	VP4	VP6	VP1	VP2	VP3	NSP1	NSP2	NSP3	NSP4	NSP5
RV2088	Genotype constellation	G1	P[4]	I2	R2	C2	M2	A2	N2	T2	E2	H2
	Open Reading frame length	981	2359	1194	3267	2658	2520	1349	954	933	528	603
	Reads mapped to contig	10,387	43,705	11,234	55,613	94,857	61,850	5640	90,290	12,856	8750	9140
RV2106	Genotype constellation	G12	P[4]	I1	R2	C2	M2	A2	N1	T2	E1	H2
	Open Reading frame length	981	2359	1194	3267	2658	2520	1349	954	933	528	603
	Reads mapped to contig	62,513	157,158	45,271	147,115	73,591	87,521	14,769	58,744	23,951	29,568	35,281
RV2150	Genotype constellation	G12	P[8]	I1	R1	C1	M1	A1	N1	T1	E1	H1
	Open Reading frame length	981	2359	1194	3267	2658	2520	1349	954	933	528	603
	Reads mapped to contig	5438	92,617	12,771	21,755	31,461	42,856	7301	88,371	15,846	183,160	31,793

Grey accent represented Wa genogroup (1). White represented the DS-1 genogroup (2).

## Data Availability

The data from this study is available on request from the corresponding author. The data are not publicly available due to policy restrictions on institutional data publication. The ORFs of sequences for all 11 genes of the breakthrough strains were deposited in the GenBank under the accession numbers (OR338245-OR338277).

## Supplementary Materials

The following supporting information can be downloaded at: https://www.mdpi.com/article/10.3390/vaccines11121759/s1, Supplementary Data: Identity matrices; Figure S1: Maximum likelihood phylogenetic tree between the VP1 gene of the Zambian strains as well as global strains; Figure S2: Maximum likelihood phylogenetic tree between the VP2 gene of the Zambian strains as well as global strains; Figure S3: Maximum likelihood phylogenetic tree between the VP3 gene of the Zambian strains as well as global strains; Figure S4: Maximum likelihood phylogenetic tree between the VP6 gene of the Zambian strains as well as global strains; Figure S5: Maximum likelihood phylogenetic tree between the NSP1 gene of the Zambian strains as well as global strains; Figure S6: Maximum likelihood phylogenetic tree between the NSP2 gene of the Zambian strains as well as global strains; Figure S7: Maximum likelihood phylogenetic tree between the NSP3 gene of the Zambian strains as well as global strains; Figure S8: Maximum likelihood phylogenetic tree between the NSP4 gene of the Zambian strains as well as global strains; Figure S9: Maximum likelihood phylogenetic tree between the NSP5 gene of the Zambian strains as well as global strains.
