# Supplementary material for: Characterization of Rotavirus Strains Responsible for Breakthrough Diarrheal Diseases among Zambian Children Using Whole Genome Sequencing
Source: Vaccines (Basel). 2023 Nov 26;11(12):1759. doi: 10.3390/vaccines11121759 (PMC10748035; doi:10.3390/vaccines11121759)
Supplement: Supplementary file 1 [file vaccines-11-01759-s001.zip › Supplementary Figures .pdf]

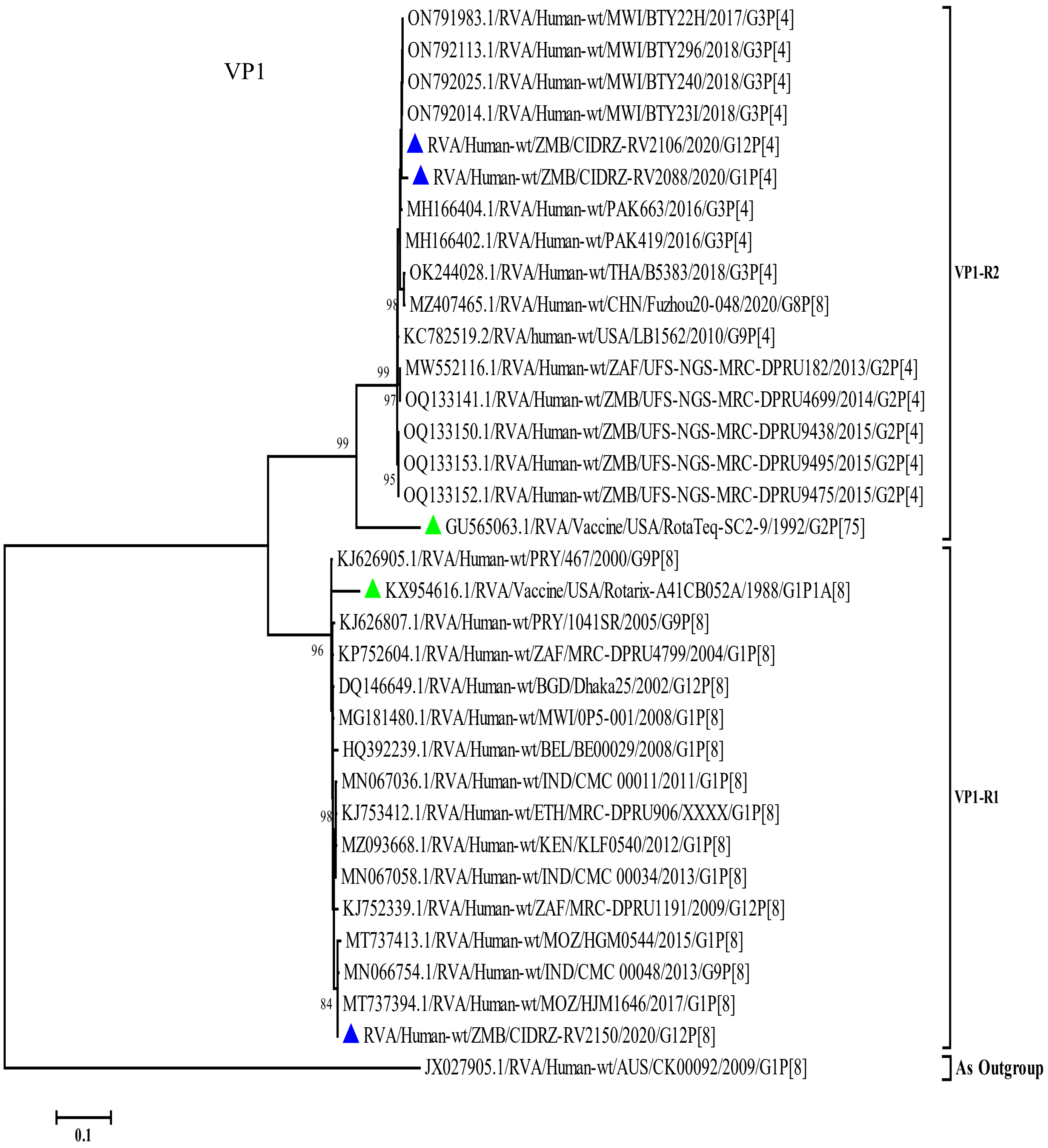

Figure S1
Maximum likelihood phylogenetic tree between the VP1 gene of the Zambian strains as well as global strains. Green filled triangles represented vaccine sequences whereas Zambian strains Blue filled triangles. Scale at the bottom indicates nucleotide substitutions per site whereas bootstrap values greater than or equal to 70 were shown on the branch nodes

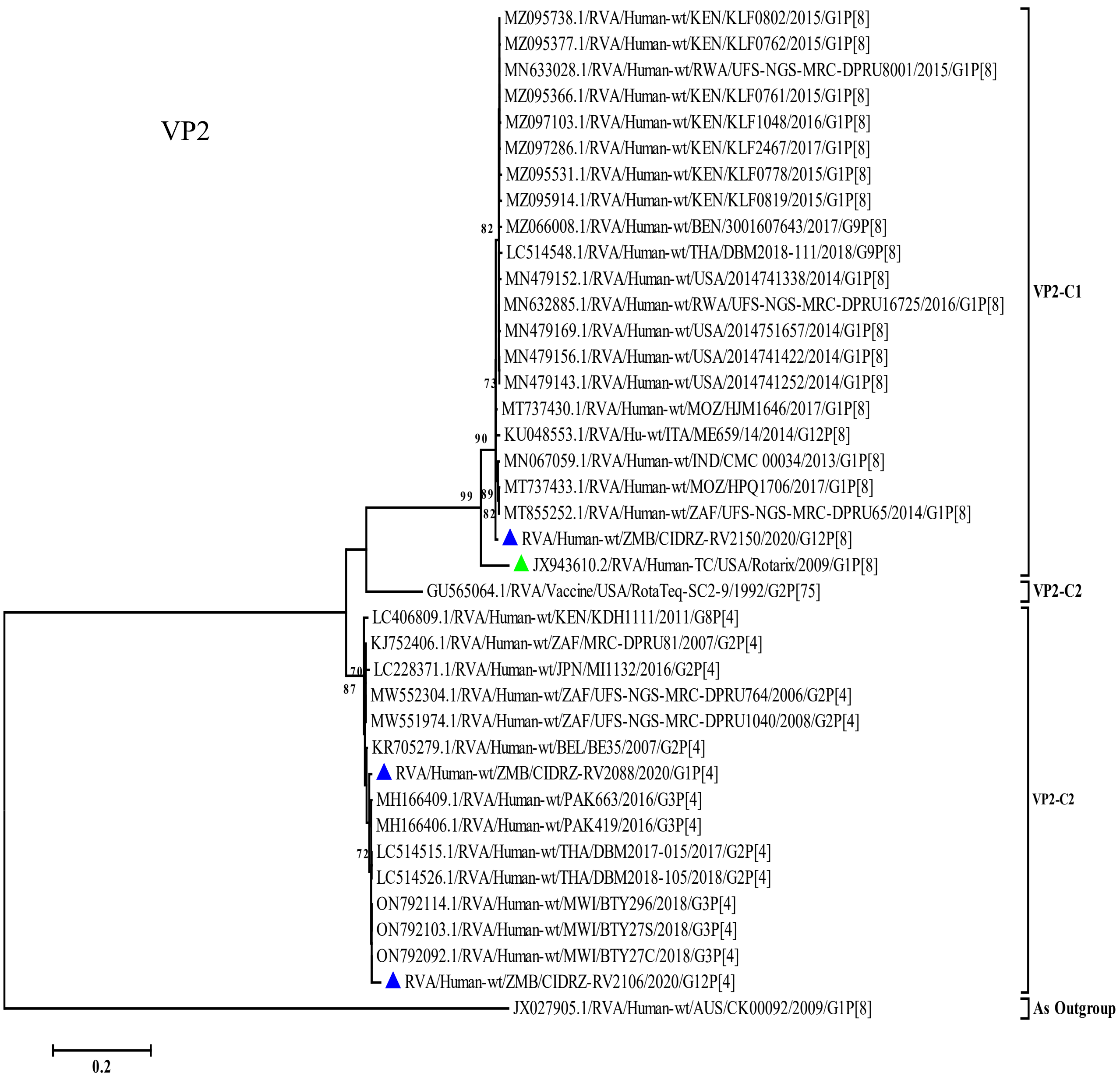

Figure S2

Maximum likelihood phylogenetic tree between the VP2 gene of the Zambian strains as well as global strains. Green filled triangles represent vaccine sequences whereas Zambian strains Blue filled triangles. Scale at the bottom indicates nucleotide substitutions per site whereas bootstrap values greater than or equal to 70 were shown on the branch nodes

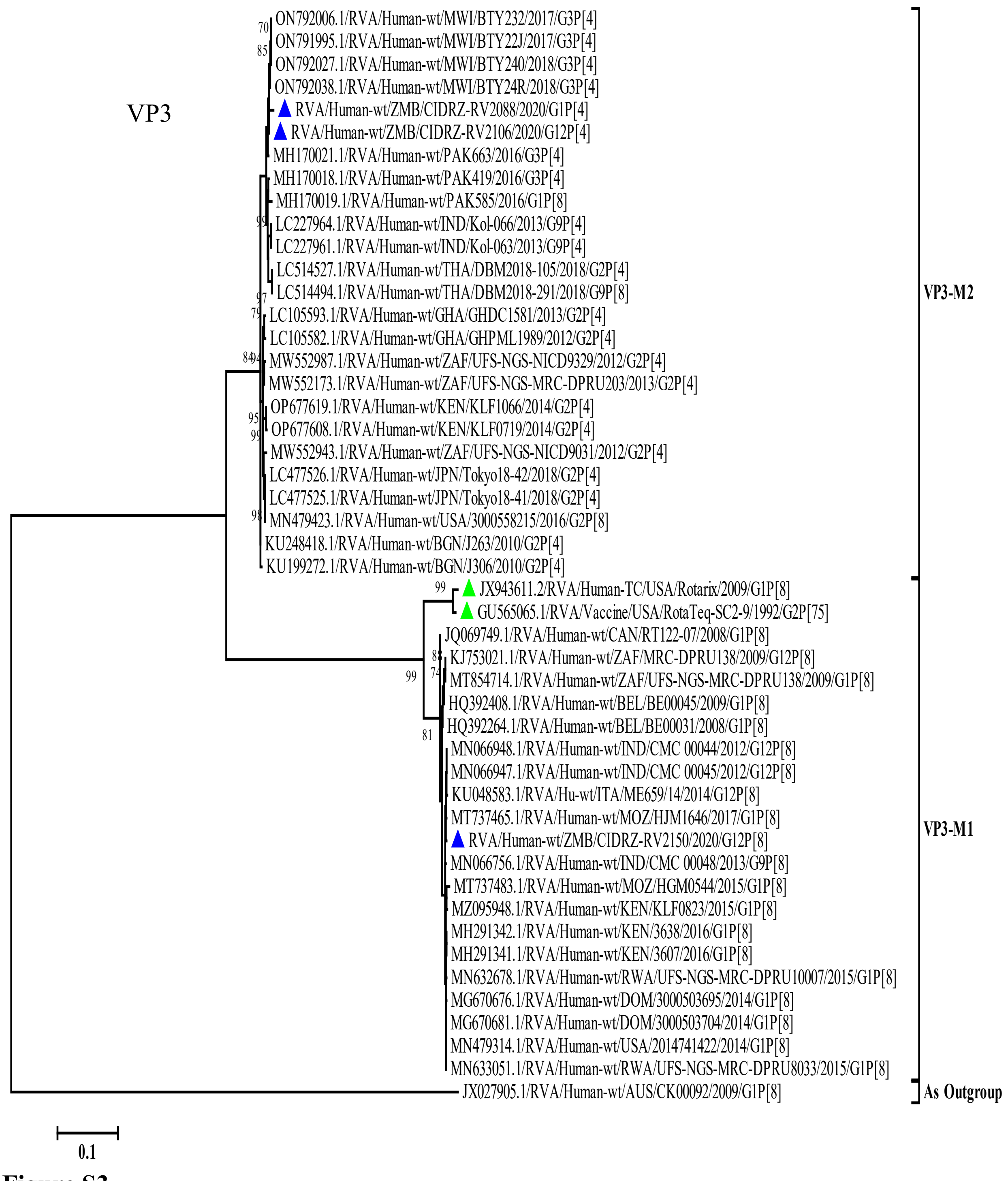

Figure S3

Maximum likelihood phylogenetic tree between the VP3 gene of the Zambian strains as well as global strains. Green filled triangles represent vaccine sequences whereas Zambian strains Blue filled triangles. Scale at the bottom indicates nucleotide substitutions per site whereas bootstrap values greater than or equal to 70 were shown on the branch nodes

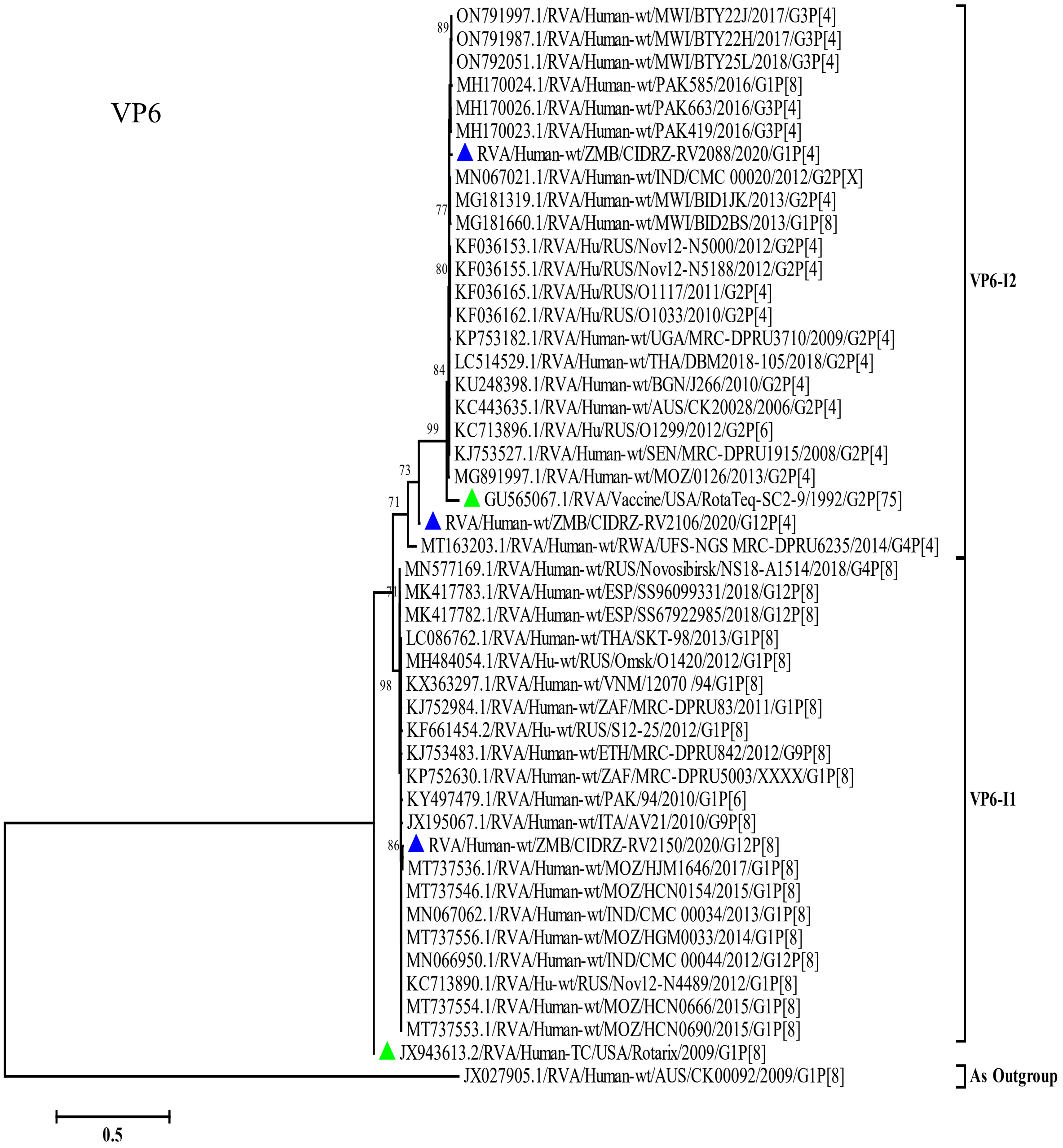

Figure S4

Maximum likelihood phylogenetic tree between the VP6 gene of the Zambian strains as well as global strains. Green filled triangles represent vaccine sequences whereas Zambian strains Blue filled triangles. Scale at the bottom indicates nucleotide substitutions per site whereas bootstrap values greater than or equal to 70 were shown on the branch nodes

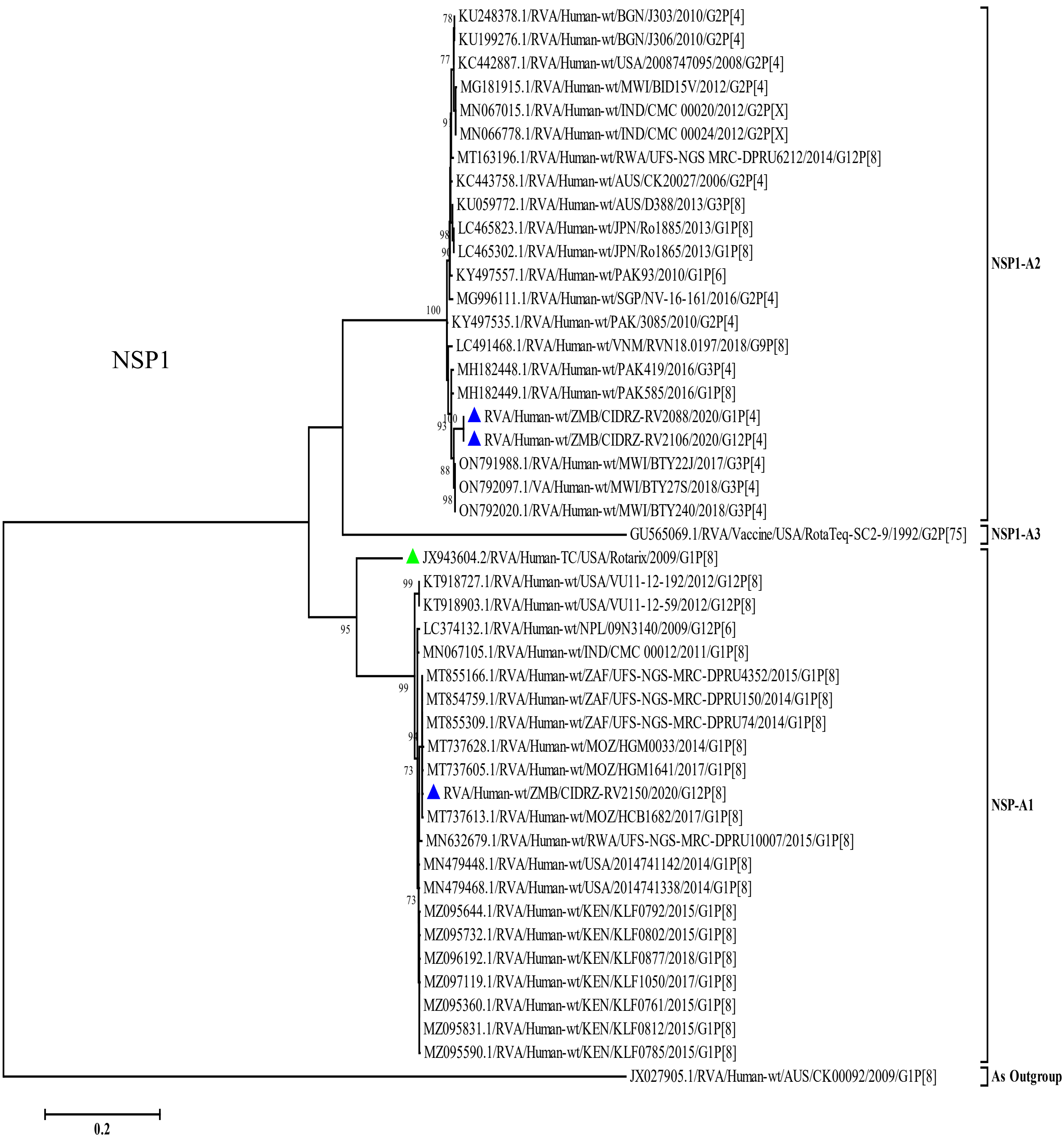

Figure S5

Maximum likelihood phylogenetic tree between the NSP1 gene of the Zambian strains as well as global strains. Green filled triangles represent vaccine sequences whereas Zambian strains Blue filled triangles. Scale at the bottom indicate nucleotide substitutions per site whereas bootstrap values greater than or equal to 70 were shown on the branch nodes

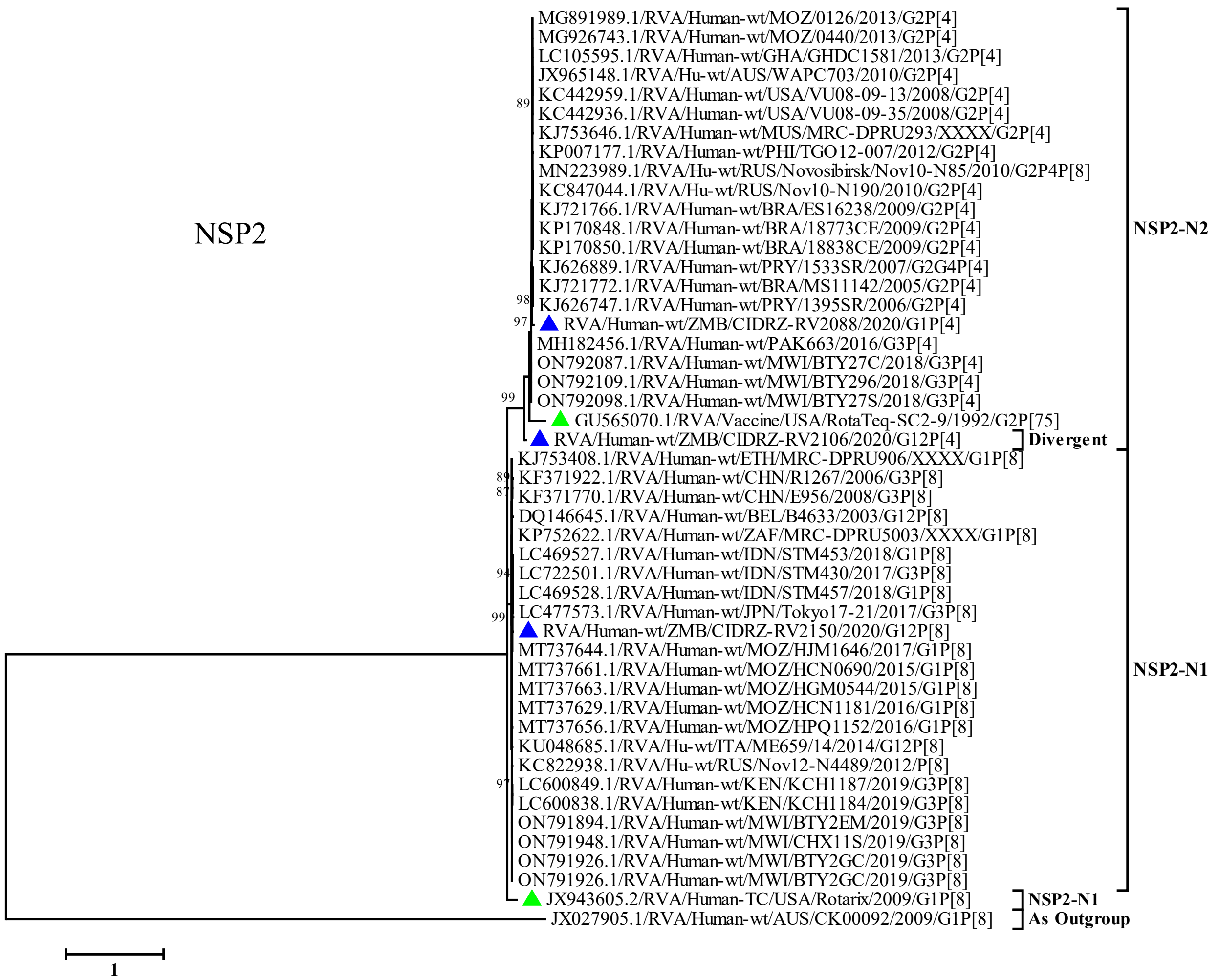

Figure S6

Maximum likelihood phylogenetic tree between the NSP2 gene of the Zambian strains as well as global strains. Green filled triangles represent vaccine sequences whereas Zambian strains Blue filled triangles. Scale at the bottom indicates nucleotide substitutions per site whereas bootstrap values greater than or equal to 70 were shown on the branch nodes

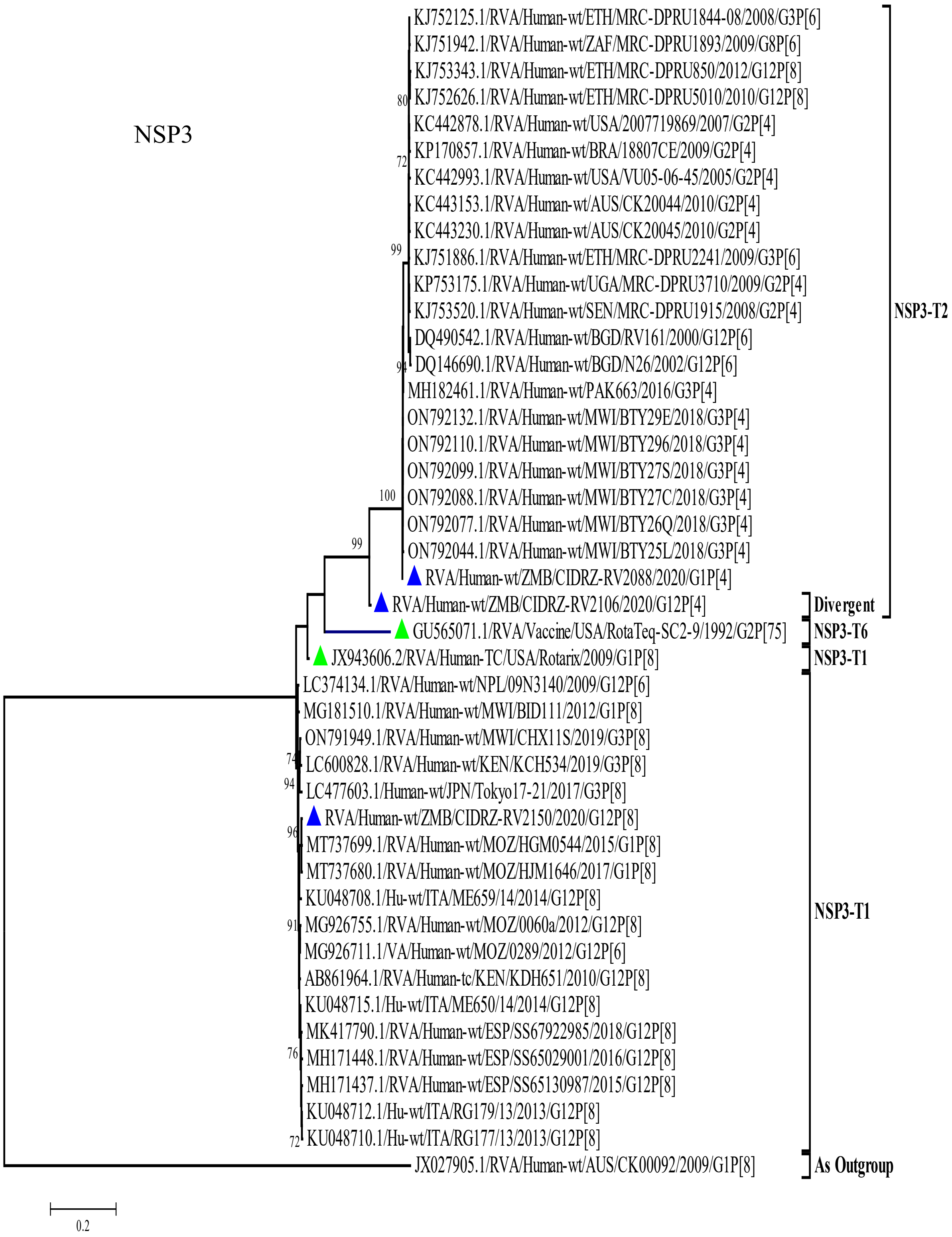

Figure S7

Maximum likelihood phylogenetic tree between the NSP3 gene of the Zambian strains as well as global strains. Green filled triangles represent vaccine sequences whereas Zambian strains Blue filled triangles. Scale at the bottom indicates nucleotide substitutions per site whereas bootstrap values greater than or equal to 70 were shown on the branch nodes

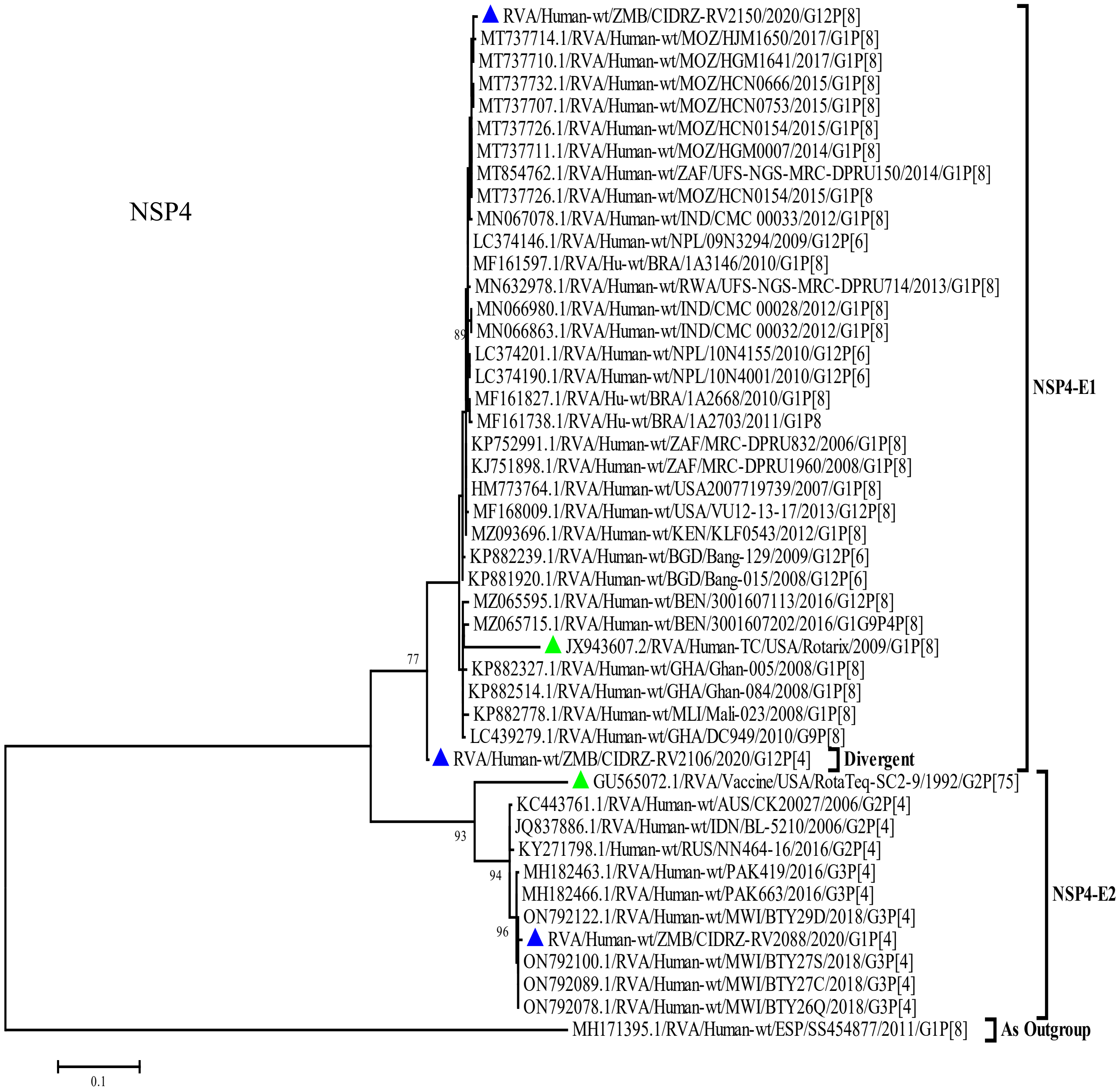

Figure S8

Maximum likelihood phylogenetic tree between the NSP4 gene of the Zambian strains as well as global strains. Green filled triangles represented vaccine sequences whereas Zambian strains Blue filled triangles. Scale at the bottom indicates nucleotide substitutions per site whereas bootstrap values greater than or equal to 70 were shown on the branch nodes

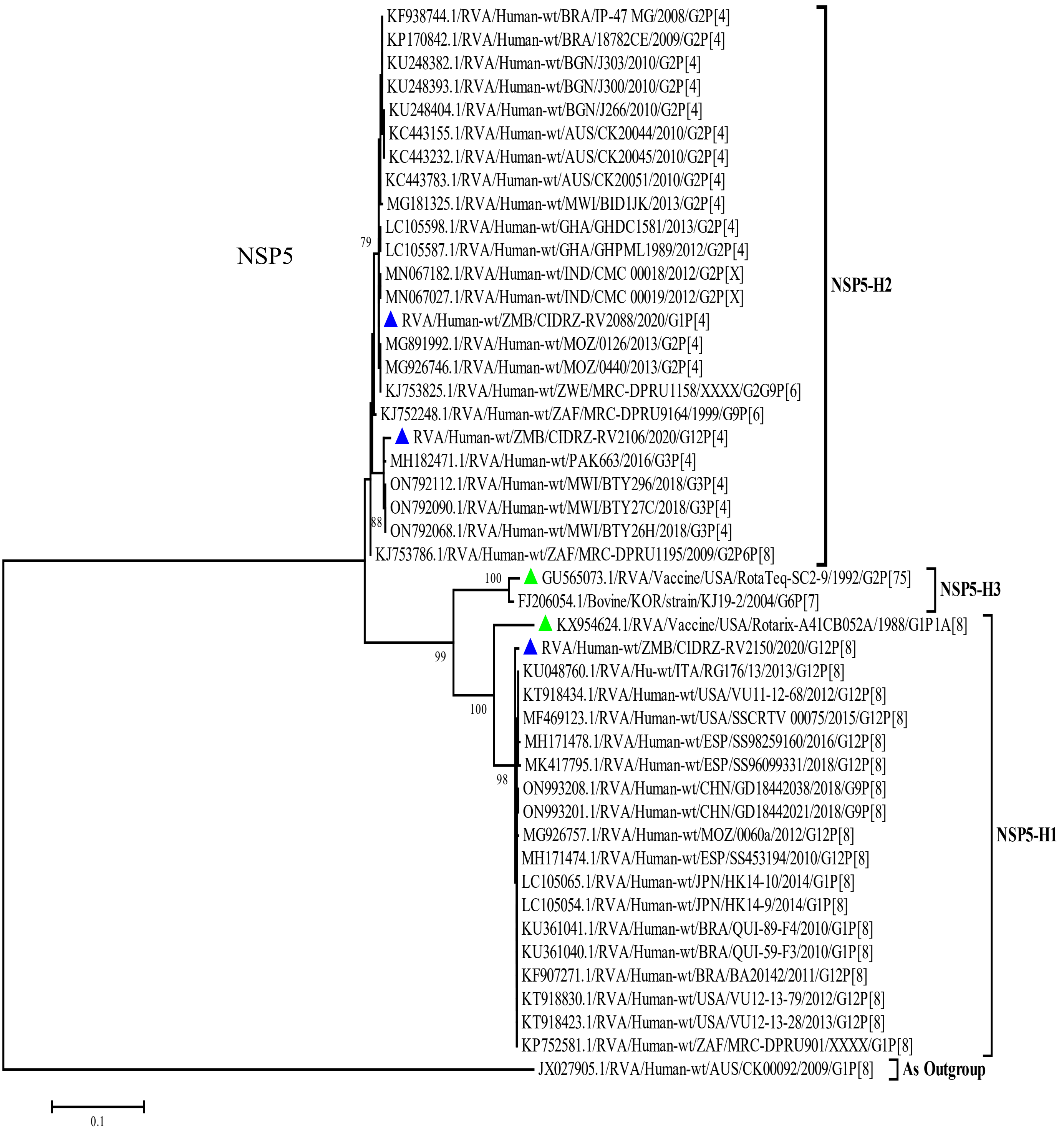

Figure S9

Maximum likelihood phylogenetic tree between the NSP5 gene of the Zambian strains as well as global strains. Green filled triangles represented vaccine sequences whereas Zambian strains Blue filled triangles. Scale at the bottom indicates nucleotide substitutions per site whereas bootstrap values greater than or equal to 70 were shown on the branch nodes
